# *In vitro* Generation of Cytotoxic T Cells With Potential for Adoptive Tumor Immunotherapy of Multiple Myeloma

**DOI:** 10.3389/fimmu.2019.01792

**Published:** 2019-08-02

**Authors:** Wafaa S. Khalaf, Mamta Garg, Yehia S. Mohamed, Cordula M. Stover, Michael J. Browning

**Affiliations:** ^1^Department of Infection, Immunity and Inflammation, Leicester University, Leicester, United Kingdom; ^2^Department of Microbiology and Immunology, Faculty of Pharmacy, Al-Azhar University, Cairo, Egypt; ^3^Department of Haematology, Leicester Royal Infirmary, University Hospitals of Leicester NHS Trust, Leicester, United Kingdom; ^4^Department of Microbiology, Imam Abdulrahman Bin Faisal University, Dammam, Saudi Arabia; ^5^Department of Immunology, Leicester Royal Infirmary, University Hospitals of Leicester NHS Trust, Leicester, United Kingdom

**Keywords:** cancer, myeloma, vaccine, immunotherapy, cytotoxic T lymphocyte

## Abstract

Multiple myeloma is a life-threatening hematological malignancy, which is rarely curable by conventional therapies. Immunotherapy, using tumor antigen-specific, cytotoxic T-lymphocytes, may represent an alternative or additional treatment for multiple myeloma. In this study, we used hybrid cell lines, generated by fusion of an EBV B-lymphoblastoid cell line (B-LCL) and myeloma cells, to stimulate *in vitro* peripheral blood lymphocytes (PBLs) from patients with multiple myeloma. We investigated induction of antigen-specific, cytotoxic T-lymphocytes to the well-defined tumor associated antigens (TAAs) hTERT, MUC1, MAGE-C1 and CS1, which have been shown to be expressed in a high proportion of cases of multiple myeloma. HLA-A2-peptide pentamer staining, interferon-γ and perforin ELISpot assays, as well as cytotoxicity assays were used. Following several rounds of *in vitro* stimulation, the hybrid cell lines induced antigen-specific, cytotoxic T-lymphocytes to four candidate TAAs in PBLs from HLA-A2^+^ multiple myeloma patients, using known HLA-A2 restricted peptide epitopes of the TAAs. In contrast, the HLA-A2^+^ myeloma cell line U266 failed to induce antigen-specific, cytotoxic T-lymphocytes *in vitro*. Our data indicate that B-LCL/myeloma hybrid cell lines induce antigen-specific, cytotoxic T-lymphocytes in PBLs isolated from multiple myeloma patients *in vitro* and may represent a novel strategy for use in adoptive immunotherapy of multiple myeloma.

## Introduction

Using conventional therapies, multiple myeloma (MM) is an incurable condition. Adoptive T cell therapy, drawing on allogeneic or autologous tumor-specific cytotoxic T-lymphocytes (CTL), may represent a potential additional treatment for MM. Hybrid cell lines, generated by fusion of professional antigen presenting cells (APC) with tumor cells, make for a promising strategy for tumor immunotherapy (1, 2). Such cell lines express a range of tumor associated antigen (TAAs) and present these in the context of both HLA class I and HLA class II molecules, with appropriate costimulatory signals (1, 2). The use of allogeneic APCs in hybrid cell formation may lead to the expression of allogeneic MHC class I aside from MHC class II by the generated hybrid cells, which exerts a desirable adjuvant effect on the stimulation of antigen-specific T lymphocytes (3).

The EBV B-lymphoblastoid cell line, HMy2, represents an appropriate APC partner for the generation of hybrid cell lines for use in tumor immunotherapy, in that it expresses HLA class I and class II, and the T cell co-stimulatory molecules, CD80 and CD86 (4, 5). We have shown that HMy2 cells grow continuously in cell culture, are sensitive to hypoxanthine-aminopterin-thymidine (HAT) and resistant to ouabain, allowing for double chemical selection of the hybrid cells after fusion (6–8). HMy2 can therefore be used as a substitute for dendritic cells (DC) as APC partner in hybrid cell formation (1, 9). The use of DC hybrid cells in treatment of various hematological and solid tumors has been successful but their clinical use is hampered, as we have outlined (10, 11): DC hybrids show low ability for replication, are short lived, the number of DCs in blood is low, and protocols to generate DC for immunotherapy are labor-intensive. A major concern of self-peptide(s) pulsed DCs is the presence of tolerance against the self-peptide(s), as they have already been presented to the immune system during disease progression. The hybrid cell lines we have generated by fusion of HMy2 and both solid and hematological tumor cells multiplied continuously in tissue culture, expressed high levels of surface molecules typical of APCs, and expressed a range of immunogenic TAAs (6–8, 10, 11). They induced T cell proliferation in mixed lymphocyte reactions *in vitro*, which we showed was dependent on the expression of the costimulatory molecules CD80 and CD86. The effector T cells induced by the hybrid cell lines recognized and killed the parent tumor cells in ^51^Cr release cytotoxicity assays (6–8). Importantly, peripheral blood lymphocytes (PBLs) isolated from healthy HLA-A2^+^ individuals and stimulated *in vitro* using the hybrid cell lines, generated CTLs with antigen specificity for several TAAs, including NY-ESO-1, survivin, MAGE-A1, and WT-1 (10, 11).

The present study aimed to progress our previous work (1, 6–8, 10, 11) and investigate the ability of hybrid cell lines, produced by fusion of HMy2 cells and myeloma cells, to induce HLA-A2-restricted tumor antigen-specific cytotoxic T cells (ASCTL) *in vitro*, in PBLs from patients with multiple myeloma.

## Materials and Methods

### The Cell Lines

HMy2 (an EBV-transformed B-LCL (8); a gift from Professor Alan Rickinson, University of Birmingham, UK) were used as APC fusion partner. The hybrid cell lines HRC and HU266 were generated by fusion of HMy2 with myeloma cells derived from patient RC, and the myeloma cell line U266, respectively. The generation of myeloma hybrid cells lines by fusion with HMy2 has been described previously (7). Briefly, HMy2 and myeloma cells were mixed 1:1, spun, and the cell pellet was treated with 1 ml 50% (w/v) polyethylene glycol / dimethyl sulphoxide (Sigma-Aldrich, UK) at 37°C for 1 min to induce cell fusion. The cells were then washed, resuspended in RPMI 1640 with 20% (v/v) fetal calf serum and cultured in a humidified atmosphere at 37°C, 5% CO_2_. After 24 h incubation, selection medium containing HAT and ouabain was added to select for hybrid formation and growth. Fusion efficiency was not formally assessed; however, the protocol has reproducibly resulted in the generation of selection resistant cell lines, and the emerging cell lines were subjected to phenotypic and genetic analysis to confirm their hybrid nature as described (7). HMy2, the hybrid cell lines HRC and HU266, and U266 (myeloma cell line; ECACC cat no 85051003) were used as stimulators of T cell responses *in vitro*. T2 cells (12) (a gift from Professor Robert Rees, University of Nottingham, UK) were used as target cells, after pulsing with the cognate TAA-derived peptides, in antigen specific IFNγ and perforin release assays, and cytotoxicity assays. K562 (a gift from Professor Sir Andrew McMichael, University of Oxford, UK), due to their lack of MHC class I expression, were used as target for assessment of NK cell activity in the cultures (13).

### Patient-Derived Primary T Cells

Samples of peripheral blood were obtained from 9 patients with MM (Supplementary Table 1). Five of the patients were HLA-A2^+^, and four were HLA-A2^−^. Peripheral blood mononuclear cells (PBMCs) were separated from the blood samples using Ficoll Paque Plus (GE Healthcare, UK).

### Phenotypic Characterization

Phenotypic characterization of the hybrid cell lines, HMy2 and U266 was carried out at least twice using direct immunofluorescent staining with FITC or PE labeled anti-HLA class I, CD80, CD138 (Beckman Coulter, UK), HLA-A2, CD86, CD19 (BD Pharmingen, UK) and HLA class II (DAKO, UK) monoclonal antibodies. Fluorescently conjugated isotype control antibodies were used as negative controls. The analysis was done using FACS Canto flow cytometer (Becton Dickinson, UK), and data were analyzed using FACS Diva software (Becton Dickinson, UK).

### Detection of TAA Proteins

#### Detection of CS1, MUC1, and h TERT Proteins by Flow Cytometric Analysis

CS1 surface expression was determined by flow cytometric analysis after staining cells, adjusted to 1 × 10^6^ cells/ ml, with 10 μl of PE-labeled anti-CS1 monoclonal antibody (mAb) or isotype control mAb (Abcam, UK).

MUC1 protein expression was assessed by indirect staining with 10 μl of anti-MUC1 mouse monoclonal antibody (anti-CD227; Insight Biotechnology Ltd; or isotype control mAb), followed by 10 μl of the PE-labeled anti-mouse IgG secondary antibody (Sigma Aldrich, UK) at the recommended dilution (1:500).

To detect intracellular human telomerase reverse transcriptase antigen (h TERT), cells were permeabilized using Fix and Perm kit (Life Technology, UK), then stained with 10 μl of FITC-labeled anti-h TERT antibody (Bioss, UK; bs-1411R) or its IgG isotype control (Bioss bs-0295P).

#### Western Blotting for Detection of MAGE-C1

Total cell proteins were extracted by lysis of 1 × 10^6^ cells in 200 μl 2X lysis buffer (Sigma Aldrich, UK). Protein lysates were separated on 8% polyacrylamide gels (SDS-PAGE) and electro-transferred to polyvinylidene difluoride (PVDF) membranes (Sigma Aldrich, UK). The membrane was blocked and incubated overnight at 4°C with rabbit anti-MAGE-C1 polyclonal antibody (Fisher Scientific, UK) at a dilution of 1: 1,000. After washing, the membrane was incubated with swine anti-rabbit immunoglobulin-HRP (DAKO, Denmark) for 1 h at room temperature. Finally, the membrane was washed and developed using an enhanced chemiluminescence (ECL) detection system, then visualized using autoradiography films.

### *In vitro* Induction of Long-Term Activated Tumor Antigen-Specific Cytotoxic T Cell Cultures by the Hybrid Cell Lines

Long-term activated T cell cultures were produced by co-culturing PBMCs isolated from patients with MM together with stimulator cell lines for several successive, weekly rounds of stimulation. The stimulator cell lines were treated with 50 μg/ml Mitomycin-C (Sigma, UK). Cells were maintained in the presence of 1 ng/ml rhIL-7 (BD Bioscience, UK), 300 U/ml rhIL-2 (Prospec, Protein-Specialists) and 10 ng/ml rhIL-15 (Prospec, Protein-Specialists) in 6-well plates, and all cultures were stimulated weekly for 4–10 weeks, at a ratio of 2 responder cells: 1 stimulator cell.

A representative example of one experiment of numbers of PBMCs that were plated, and in the 1st, 2nd, 3^rd^, and 4th stimulation is shown in Supplementary Table 2. We examined the presence of antigen specific response in the culture in the third week according to the previously published protocol (11), and then again in the 4th week. Because we observed an increase in percentage of the antigen specific response in the fourth week rather than the 3rd week, and a reasonable number of stimulated CTLs was needed for further peptide-specificity and functional activity evaluation for four different peptides, a 4-week stimulation protocol was used for this work.

#### MHC Class I Pentamer Staining

Recognition and estimation of tumor antigen-specific CTLs were carried out by flow cytometric analysis of long-term activated T cells stained with FITC-labeled anti-CD8 and R-PE-labeled Pro5 MHC class I pentamers incorporating different HLA-A2 binding peptides derived from the TAAs of interest (Proimmune Limited, UK). The relevant peptide sequences were: MUC1 (12–20; LLLLTVLTV), hTERT (540–548; ILAKFLHWL), MAGE-C1 (959–968; ILFGISLREV), CS1 (239–247; SLFVLGLFL), and a negative control peptide derived from West Nile Fever virus (WNF, 294–302; LGMSNRDFL).

#### Tumor Antigen-Specific Perforin and IFNγ ELISpot Assays

To determine the antigen specific activity of the ASCTL produced in the long-term stimulated cultures, HLA-A2 restricted TAA peptide-pulsed T2 cells were used as stimulator cells in IFNγ and perforin ELISpot assays. Plates were coated with the primary antibody 18 h prior to incubation of cells, and the percentage of peptide-specific cytotoxic T cells in the cultures was determined using IFNγ and perforin ELISpot kits (Mabtech AB, Sweden). The length of culture for this study was optimized to 3 and 5 days, respectively. Peptides used to pulse T2 cells were the same as were incorporated in the MHC class I pentamers.

#### DELFIA EuTDA Cytotoxicity Assays

Antigen-specific cytotoxic activity of the produced CTL was determined using (non-radioactive) DELFIA® EuTDA Cytotoxicity Reagents (PerkinElmer Inc., UK), according to the manufacturer's instructions, using peptide pulsed T2 cells as target cells, and over a range of effector cell: target cell ratios. Peptides used to pulse T2 cells were the same as were incorporated in the MHC class I pentamers. After incubation for 2 h, the released fluorescence was measured in a time-resolved fluorimeter (Horiba, UK), and the percentage specific release was calculated.

### Statistical Analysis

The statistical significance of differences of pentamer staining data was analyzed by one-way ANOVA, and the ELISpot and cytotoxicity data were analyzed by two-way ANOVA, using GraphPad Prism 7 software (GraphPad Software, San Diego, CA, USA). A statistically significant difference between parameters was considered when *p*-value was <0.05.

## Results

### Phenotypic Characterization of the Hybrid Cell Lines, HMy2, and U266

**Figure 1:**
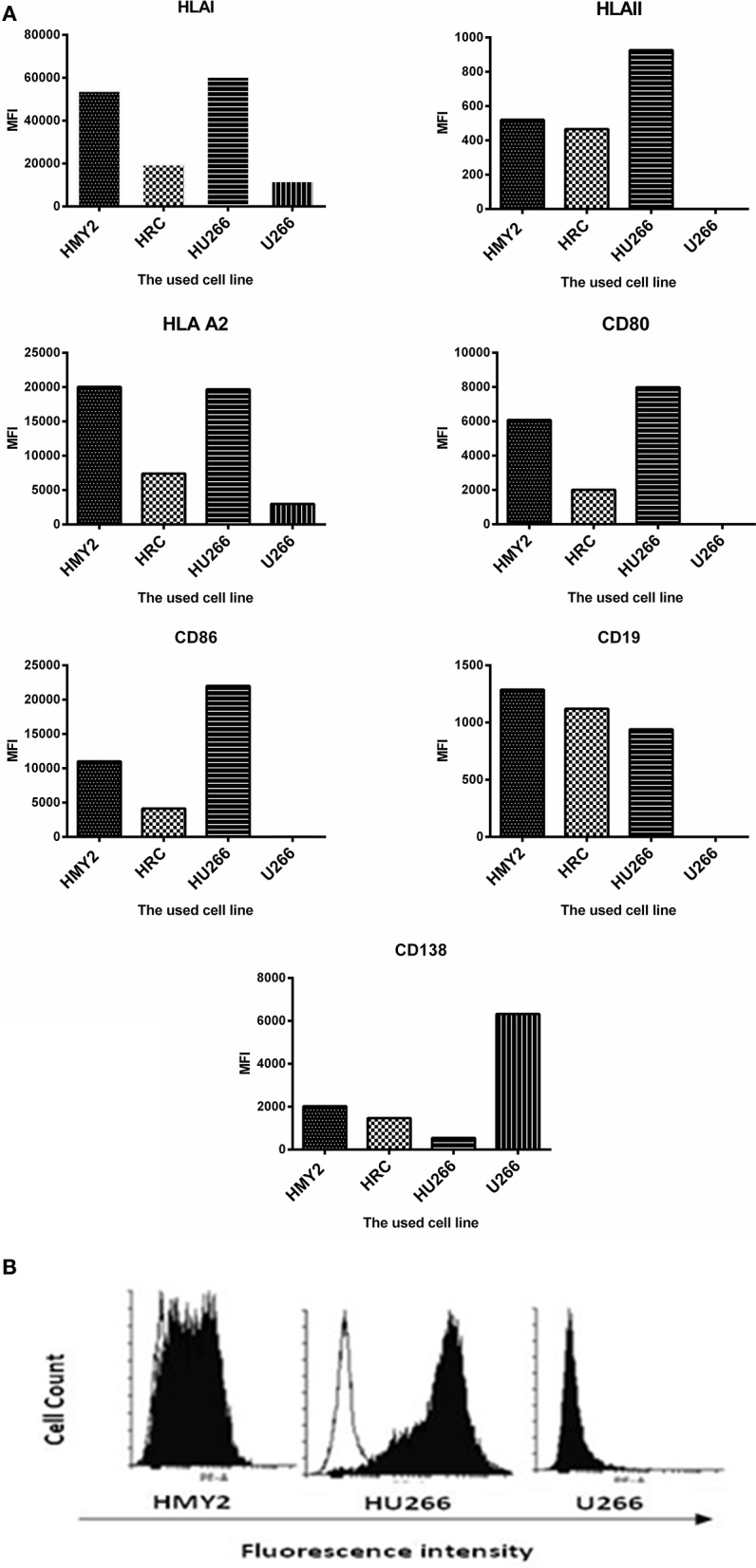
Phenotypic characterization of HMY2, the hybrid cell lines, and U266. **(A)** Mean fluorescence intensity (MFI) of FACS analysis for determination of the expression of HLA class I, HLA-A2, HLA class II, CD80, CD86, CD19, and CD138. **(B)** A representative example of flow cytometry histograms showing expression of co-stimulatory marker CD86 on HU266, the APC cell line HMY2 and the myeloma cell U266. The analyses were conducted at least twice.

Flow cytometric analysis of the expression the costimulatory molecules CD80 and CD86, lineage-specific markers CD19 and CD138, and HLA class I, class II, and HLA-A2 by the hybrid cell lines HRC and HU266, HMy2, and U266 are shown in Figure 1A. All cell lines reproducibly expressed HLA class I and HLA-A2, although at different levels. Both the APC parent (HMy2) and the hybrid cell lines expressed CD80, CD86, and HLA class II, though they were consistently absent in the myeloma cell line U266. In addition, both the hybrid cell lines and HMy2 expressed all candidate cell surface markers at a higher level than the myeloma cell line U266, with the exception of the plasma cell marker CD138 (Figure 1A). Representative histograms showing expression of CD86 by HMy2, HU266, and U266 cells are shown in Figure 1B.

**Figure 2:**
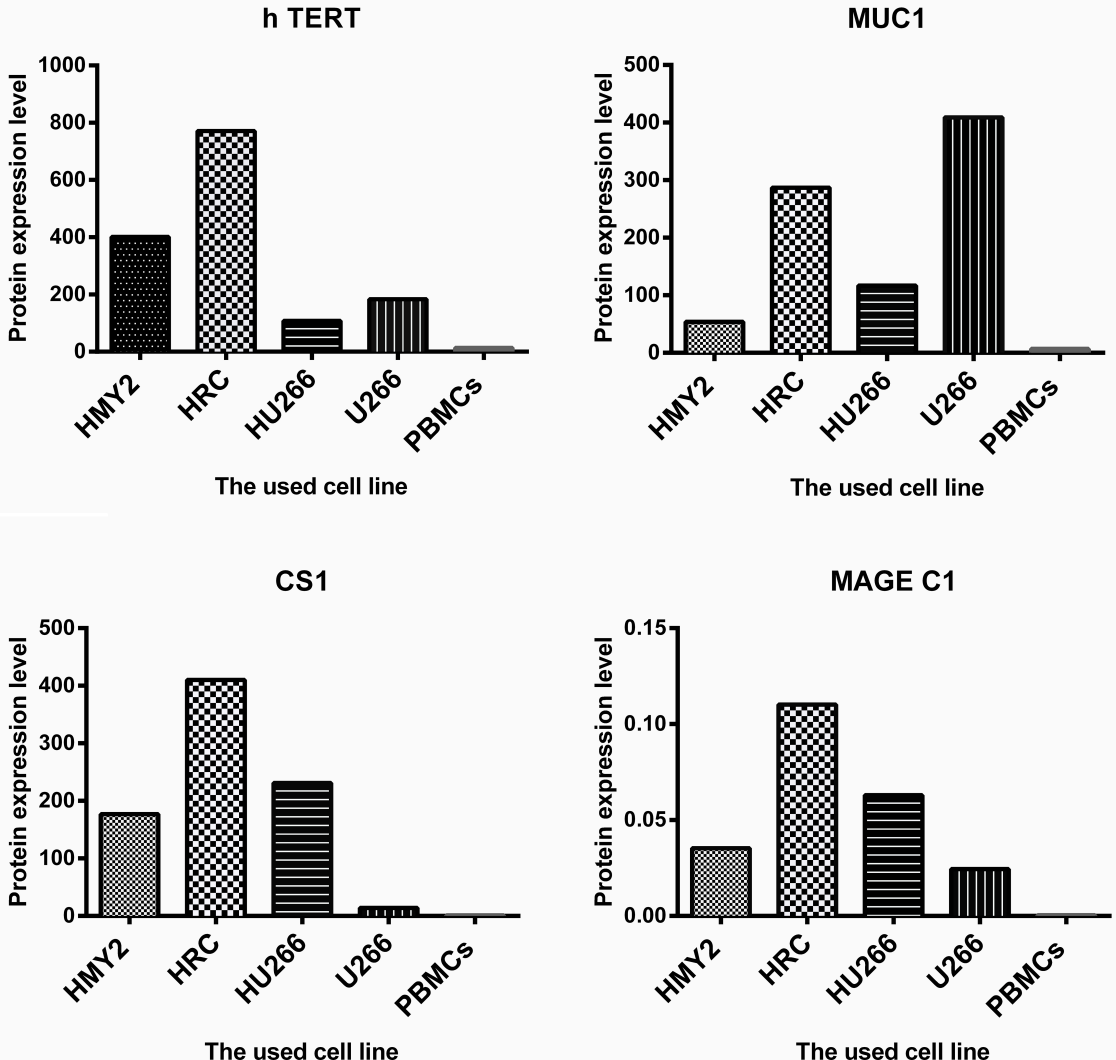
The level of protein expression of the indicated antigens by the used cell lines. For MUC1, h TERT, and CS1, the presented values are the MFI of FACS analysis (means of two). For MAGE C1, the values were determined by imaging densitometry following Western blot (mean of two). The presented MFI values are (the MFI of examined marker expression—MFI of matched isotype control).

The potential use of the hybrid cell lines as immunogens for induction of antigen specific immune response in tumor immunotherapy is determined not only by the APC-derived phenotype, but also by the expression and effective presentation of the TAAs in the context of MHC molecules. We therefore investigated the protein expression of the TAAs MUC1, hTERT, CS1, and MAGE-C1 for the hybrid cell lines, HMy2 and U266. Both hybrid cell lines expressed MUC1, hTERT, CS1, and MAGE-C1 at significantly higher levels than was seen in normal PBMCs (used as a negative control) (Figure 2). HMy2 and U266 also expressed the TAAs at higher levels than PBMCs.

These results indicated that the hybrid cell lines preserved the phenotypic characteristics of their parent APC and expressed all four candidate TAAs at the protein level.

### Induction of Antigen-Specific CTL in PBMCs of MM Patients

#### HLA-A2-Peptide Pentamer Analysis

The specificities of the induced antigen specific CTL were investigated by staining with FITC-labeled anti-CD8 and PE-labeled HLA-A2-peptide pentamers specific for hTERT, MUC1, MAGE-C1, and CS1, followed by flow cytometric analysis. Three sets of negative controls were used in the experiments to exclude the occurrence of non-specific recognition due to the remaining T cell population: The first one was an irrelevant peptide antigen, West Nile Fever virus peptide (WNF), to ensure the antigen-specific nature of the induced T cell responses. The second negative control was using stimulated PBMCs from HLA-A2^−^ MM patients, as a negative control for the HLA-A2^+^ restricted responses. The third negative control was unstimulated PBMCs from the HLA-A2^+^ MM patients, in parallel with the stimulated T lymphocytes from the same individuals. These PBMCs were also used as a baseline, to indicate successful induction of the antigen specific T lymphocyte in the stimulated cultures.

**Figure 3:**
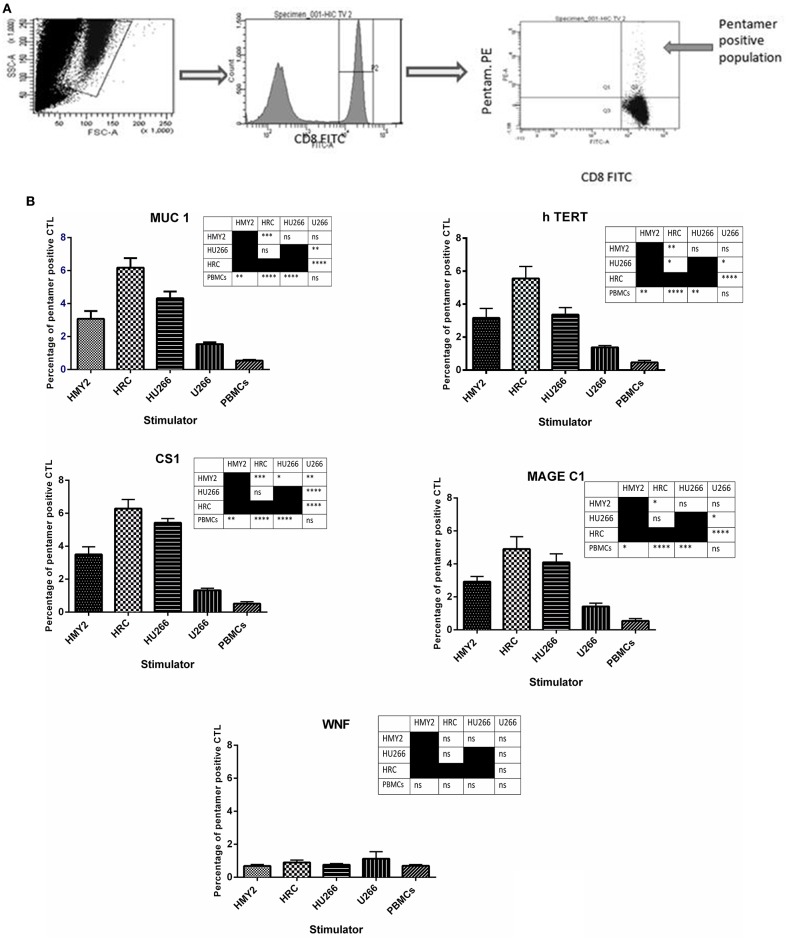
Presence of tumor antigen-specific CD8+ T cells in cultures, using HLA-A2-peptide pentamer staining. **(A)** Sequential gating on the live T cells using SSC X FSC dot plot, followed by gating the CD8+ population, and finally determining the percentage of the double positive CD8+ pentamer+ T cells on the FITC vs. PE dot plot. **(B)** Percentages of positive MUC1, h TERT, CS1, MAGE-C1, and WNF HLA-A^*^0201 restricted, HLA-A2-peptide pentamer-stained CTL. The CTL were generated by four rounds of stimulation of PBMCs isolated from five HLA-A2+ patients, using the hybrid cell lines, HRC and HU266, or the parent cell lines HMY2 and U266 as stimulators. The percentages were determined using flow cytometric analysis. Results are presented as mean ± SEM. The levels of significance have been summarized in the table next to each chart and presented as asterisks (^*^p < 0.05, ^**^p ≤ 0.01, ^***^p ≤ 0.001, and ^****^ *p* ≤ 0.0001).

As shown in Figure 3, both hybrid cell lines (HU266 and HRC) induced significantly higher levels of MUC1, hTERT, CS1, and MAGE C1 specific CD8^+^ T cells, compared with both U266 and the unstimulated PBMCs. HRC hybrid cell line produced the highest responses (significantly higher than HMy2 for each of the antigens investigated), whilst HU266 induced significantly higher pentamer-positive CS1-specific cells than HMy2 cells. HMy2 induced a greater response to each of the antigens compared with unstimulated PBMCs, but only induced a higher response than U266 for CS1. U266 failed to induce a significant increase in any of the candidate antigen specific CD8^+^ T cells, compared with unstimulated PBMCs. There was no significant elevation for any of the negative control parameters compared with the unstimulated PBMCs (Figure 3 and Supplementary Figure 1). The small percentage of pentamer positivity in PBMCs isolated from HLA-A2 negative patients may be due to mismatched HLA. However, there was no significant elevation of HLA-A2 restricted antigen specific CD8^+^ T cell percentages compared with that of the background of the unstimulated PBMCs for those patients.

#### Detection of Antigen Specific CTLs Using IFNγ and Perforin ELISPOT Assays and Cytotoxicity Assays

**Figure 4:**
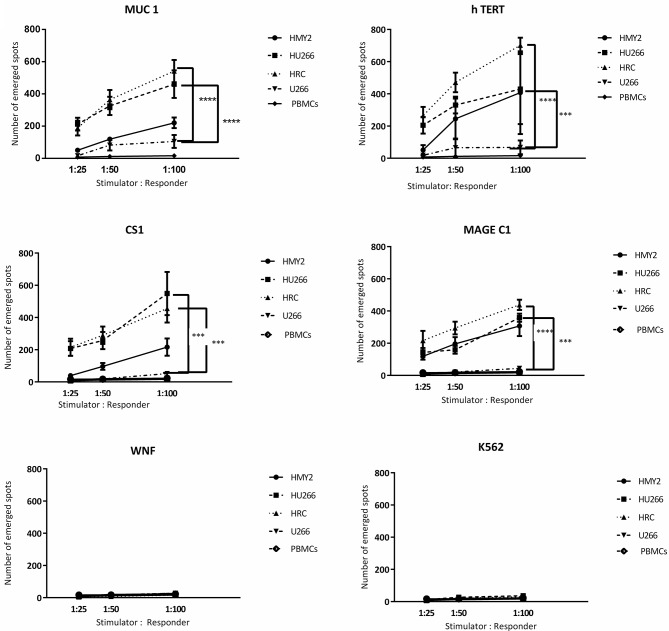
IFN-γ release ELISpot assays. Allogeneic PBMCs, isolated from five different HLA-A2+ MM patients were stimulated with HMY2, HU266, HRC, or U266. The resulting responder cells were mixed with T2 stimulator cells (after pulsing with MUC1, hTERT, CS1, MAGE-C1, and WNF derived peptides) in IFN-γ ELISpot assays. K562 were used as controls to assess NK activity. The number of emerged spots (Y axis) is presented as mean value of the patients' responses ± SEM. The stimulator: responder ratios are presented on the X axis. PBMCS stimulated with the used cell lines without further stimulation in the ELISPOT assay were used as negative controls. Levels of significance difference between the level of IFN-γ release of antigen specific T cells induced by the hybrid cell lines (HRC and HU266) and the parent myeloma cell line U266 are indicated as asterisks (^***^p ≤ 0.001 and ^****^p ≤ 0.0001).

**Figure 5:**
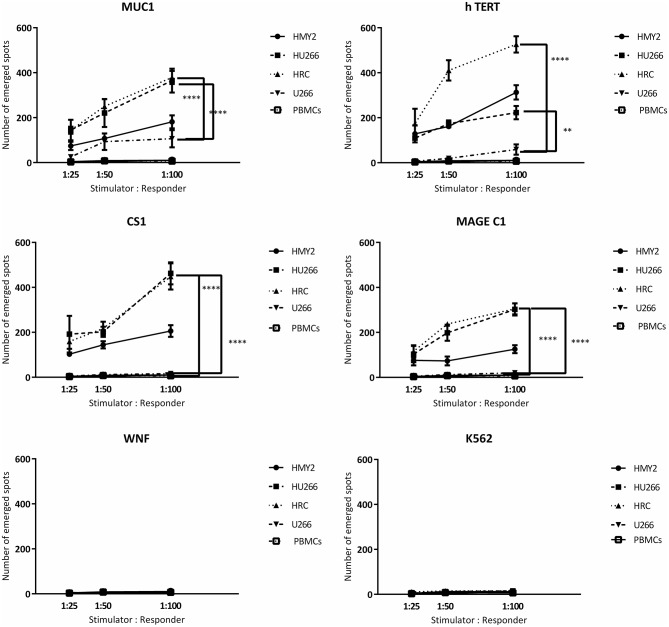
Perforin release ELISpot assays. Long-term stimulated responder cells (PBMCs, isolated from three different HLA-A2+ MM patients and stimulated with HMY2, HU266, HRC, or U266) were co-cultured with T2 stimulator cells (after pulsing with MUC1, TERT, CS1, MAGE-C1, and WNF derived peptides). K562 were used as controls to assess NK activity in perforin ELISpot assays. The number of the emerged spots (Y axis) is presented as mean value of the patients' responses ± SEM. The stimulator to responder ratios are presented on the X axis. PBMCS stimulated with the used cell lines without further stimulation in the ELISPOT assay were used as negative controls. Levels of significance difference between the level of perforin release of antigen specific T cells induced by the hybrid cell lines (HRC and HU266) and the parent myeloma cell line U266 are indicated as asterisks (^**^p ≤ 0.01 and ^****^p ≤ 0.0001).

Following the detection of antigen specific CD8^+^ T lymphocytes in the long-term stimulated cultures, we wanted to estimate their functional activity using IFNγ and perforin ELIspot, and cytotoxicity assays. IFNγ and perforin releasing responses of the stimulated T lymphocytes in ELIspot assays are shown in Figures 4, 5. T2 cells, pulsed with the same TAA-derived peptides as were used in the HLA-A2-peptide pentamers, were used as stimulators in the ELIspot assays. An irrelevant peptide (WNF peptide) was used as a negative control. Additional negative controls were K562 (to examine NK cells activation) stimulated T cells without further stimulation during the ELISpot assay (to assess the background response), and cultured, stimulated PBMCs from HLA-A2^−^ MM patients. The hybrid cell lines (HU266 and HRC) stimulated significant elevation of IFNγ and perforin releasing cells in response to restimulation with MUC1, CS1, hTERT, and MAGE C1 derived peptide-pulsed T2 cell line, compared with cultured PBMCs without further re-stimulation, the parent myeloma cell line U266, and their parent APC cell line, HMY2 (except for MAGE C1 specific IFNγ releasing response). There was no statistically significant difference in this response between HMy2 and U266 simulated cultures at most of the ratios used. HRC induced cultures showed the highest IFNγ and perforin releasing response, followed by HU266 and HMY2. The parent myeloma cell line, U266, showed the lowest IFNγ and perforin releasing responses in stimulated cultures. No significant activity was seen against the irrelevant antigen (WNF peptide) or K562 cells (Figures 4, 5) or using stimulated cultures of PBMC from HLA-A2^−^ MM patients (Supplementary Figure 2). In one HLA-A2^−^ patient sample, some IFNγ releasing response was observed in the IFNγ ELISpot assays, in response to the tumor antigenic peptides, but not to WNF peptide. Absence of this response to WNF pulsed T2 cells suggested a lack of allogeneic (mismatched HLA) response to T2 cells. In addition, absence of HLA-A2 expression in this patient suggested that the response observed was an allogeneic response to HLA-A2, rather than an antigen-specific HLA-A2 restricted response (Supplementary Figure 3). One possible explanation for the IFNγ ELISpot results was that the presence of some antigenic peptides stabilized the HLA-A2 molecule on the surface of T2 cells more effectively than others, which may lead to upregulation of HLA-A2 expression at the cell surface, and induction of an HLA-A2- specific allogeneic response. To pursue this further, we examined the actual stability of HLA-A2 expression of the T2 cell line after pulsing with all the candidate peptides, using the flowcytometric analysis. Unpulsed T2 cells were used as a negative control. The results showed that MUC1 and h TERT derived peptides caused the highest level of HLA-A2 stabilization, followed by CS1 and MAGEC1, whilst WNF virus derived peptide caused lowest HLA-A2 stabilization level. Moreover, Syfpeithi score software (www.syfpeithi.de) was used to estimate the binding avidity of each HLA-A2 loaded peptide of the used MM antigen derived peptides and WNF derived peptide. The results indicated that the TAA have a relatively high binding avidity for HLA-A2, compared with WNF virus peptide by both level of HLA-A2 expression and Syfpeithi score. The predicted HLA-A2 binding avidity of MUC1 was the highest followed by h TERT, CS1, MAGEC1, and WNF in descending order. Accordingly, these data suggest that the increase of HLA-A2 stability caused by the TAA peptide could lead to some HLA-A2 specific allogeneic response in these particular cultures, using PBMCs from the HLA-A2^−^ myeloma patients.

**Figure 6:**
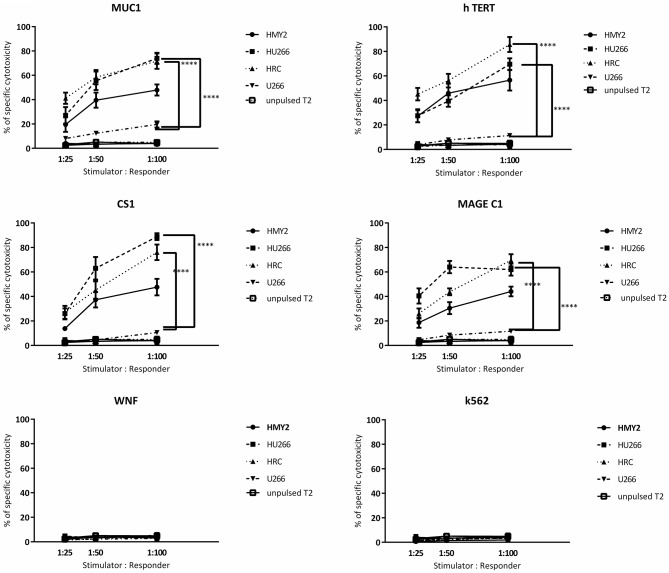
Europium release cytotoxicity assays. Individual plots show the percentages of specific lysis (Y axis) of peptide pulsed T2 cells, by T cells from cultures stimulated by the hybrid cell lines (HRC and HU266), HMy2 cells, and the parent myeloma cell line U266, from five different HLA-A2+ MM patients. The stimulator to responder ratios are presented on the X axis. The peptides used to pulse T2 cells are the same as those used in ELISPOT assays shown in Figures 3, 4. K562 were used as controls to assess NK activity. Data are presented as mean ± SEM of % specific lysis induced, and statistically significant differences are indicated as asterisks (^****^p ≤ 0.0001).

Regarding the T cell-mediated cytotoxicity assays, HRC and HU266 induced cultures showed the highest cytotoxic responses against the candidate antigens (peptide-pulsed T2 cells compared with unpulsed T2 cells) (Figure 6). HMy2 also induced antigen-specific cytotoxicity in most of the assays, but to a lesser extent than the hybrid cell lines. In contrast, U266 failed to induce specific cytotoxic responses to any of the antigens, compared with unstimulated PBMCs from the same individuals. No significant activity was seen against the irrelevant antigen (WNF peptide) or K562 cells in any of the cultures (Figure 6) or using stimulated cultures of PBMC from HLA-A2^−^ MM patients (Supplementary Figure 4).

## Discussion

In this paper, we have shown that *in vitro* co-culture of PBMCs (isolated from patients with myeloma) with hybrid cell lines (generated by fusion of the APC cell line HMy2 with myeloma tumor cells) resulted in the induction and expansion of tumor antigen-specific T cells with cytotoxic and IFN-γ-releasing activity against a range of relevant TAAs expressed by myeloma cells (MUC1, hTERT, CS1, MAGE C1). Such antigen-specific T cells, if produced to GMP standards, could have potential for use in adoptive cellular immunotherapy of multiple myeloma. The use of T cells in tumor immunotherapy has long been of interest, based on their high degree of tumor specificity, ability to expand clonally to high numbers, potential for memory, and trafficking properties (14). The potential use of immunotherapy for multiple myeloma, however, must be considered in the context of an immunosuppressive bone marrow microenvironment that promotes myeloma cell survival and progressive immune dysregulation (15). Some authors have reported unresponsiveness of T cells isolated from MM patients to tumor cells (16, 17). Our data indicate that this unresponsiveness can be overcome, and support other reports, which have shown enhanced cytotoxic responses of T cells to myeloma tumor cells *in vivo* following tumor antigen vaccination, or *ex vivo* activation of myeloma-specific T lymphocytes (18–20).

Adoptive cellular immunotherapy, using autologous or allogeneic antigen specific CTL, has been shown to be of clinical benefit in the treatment of patients with various hematological malignancies, including multiple myeloma (21–24). MM is an ideal target disease for trials of treatment using adoptive T cell immunotherapy, for several reasons. MM has been considered incurable by conventional chemotherapies, so lends itself to the introduction of novel therapies. The use of allogeneic stem cell transplant in MM has induced long-lasting remission in at least a subset of patients, due to the graft-vs.-myeloma effect of antigen-specific donor-derived T cells (25). However, toxicity, due mainly to graft-vs.-host disease, has limited its utility (25). Allogeneic T cell infusions have been used in patients with myeloma in cases of relapse after allogeneic stem cell transplant, or in partial remission, which resulted in complete remissions in a number of patients (26–28). The use of antigen-specific T cells in adoptive immunotherapy may reduce graft-vs.-host disease and enhance anti-tumor effects, although issues with toxicity still need to be overcome (29). The feasibility of generating antigen specific CTL to GMP standards *in vitro* and using them in treatment of myeloma patients has been shown (23).

APC/myeloma cell hybrids have been reported previously to induce tumor specific CTLs. Hybrid cells, prepared by fusion of autologous myeloma cells and dendritic cells, induced MHC restricted myeloma-specific cytolytic T cells, which could not be obtained by using the patients' tumor cells alone or DC alone (21). Furthermore, Rosenblatt et al. (20), using dendritic cell/MM cell fusion vaccine following autologous stem cell transplantation, showed that vaccination resulted in expansion of MM-specific CD8+ T cells, and cytoreduction of minimal residual disease.

The hybrid cell lines used in this study were generated using an EBV B-LCL, HMy2, as APC, in fusion with myeloma cells. The resulting hybrid cell lines showed several phenotypic differences to the myeloma cell line, U266. Importantly for stimulation of T cell responses, levels of expression of MHC class I and class II were significantly higher, and the T cell costimulatory molecules CD80 and CD86, which were not expressed by the myeloma cell line U266, were expressed on the hybrid cell lines. In addition, the hybrid cells expressed CD19, characteristic of the parent B-lymphoblastoid cells, but did not express the myeloma marker CD138. We have observed previously the dominance of lymphoblastoid cell markers in immunostimulatory hybrid cells generated with HMy2 (7).

The generation of EBV-specific T cells upon several stimulations was ruled out in a previous experiment (6). Using similar hybrid cell lines, we have used HMy2-tumor cell hybrid for induction of PBMC from both EBV-seropositive and EBV-seronegative donors. Comparable response of lymphocyte proliferation was seen, showing that the observed stimulation was not due to presentation of EBV antigens by the hybrid cells.

For our study we selected TAAs with high prevalence in multiple myeloma, CS1, MUC1, hTERT, and MAGE C1, to assess induction of tumor-specific immune responses. These antigens are tumor specific, highly immunogenic, and potent targets for tumor immunotherapy, especially for antigen specific CD8^+^ T cells (29–39). Our data clearly indicate that HMy2-derived hybrid cell lines had the ability to induce antigen-specific CTLs in MM patient PBMCs, using HLA-A2-peptide pentamer staining, antigen-specific ELIspot and cytotoxicity assays. In contrast with the hybrid cell lines, U266 myeloma cells failed to induce antigen specific CTL populations to any of the candidate antigens, despite expressing these antigens (in some cases, at higher levels than in the hybrid cell lines). This may be due to the absence of the expression of the co-stimulatory molecules, as stimulation of T lymphocytes in absence of the secondary stimulation signals, could lead to tolerance (40), or to lack of MHC class II expression for antigen presentation to CD4+ (helper) T cells. The parent APC cell line, HMy2, also expressed the tumor antigens investigated in this study, but generally at lower levels than the hybrid cell lines or U266. We did not identify any TAAs relevant to myeloma that were not expressed by HMy2 cells. This may be due to the related nature of HMy2 (as immortalized B-lymphoblastoid cells) and myeloma (as fully differentiated tumor plasma cells). It is, however, possible that expression of some tumor antigens unique to myeloma cells was lost during the process of hybrid formation. Interestingly, non-fused HMy2 induced weaker T cell responses than the hybrid cell lines derived from fusion of HMy2 and myeloma cells, in spite of expressing the relevant TAAs. The use of K562, as a control, indicated absence of NK cell activity in the stimulated cultures. Furthermore, the absence of responses to an irrelevant (West Nile Fever virus) peptide confirmed that the observed responses were obtained from antigen specific T-lymphocytes. Finally, the MHC-restricted nature of the responses was demonstrated by the lack of induction of CTL in PBMC from HLA-A2^−^ MM patients for the antigen responses used in this study. These results suggest that the hybrid cell lines could be used to induce antigen-specific T cells for use in adoptive T cell immunotherapy, after production under GMP protocols.

We have shown that B-LCL/myeloma hybrid cell lines can be used to generate allogeneic, HLA A2 restricted, tumor antigen-specific CTL responses *in vitro*. We have demonstrated previously that hybrid cell lines like those used in this study can also generate tumor antigen-specific CTLs *in vitro* in the autologous setting (11). To date, no *in vivo* studies have been carried out using this approach. *In vivo* clinical studies are necessary as a next stage to prove hybrid cell competence in cancer immunotherapy. This could be carried out in an animal model, by adoptive transfer of CTL generated *in vitro* to immunodeficient mice previously challenged with (human) myeloma cells (41). For use in humans, the hybrid cell lines and the T cell cultures would have to be carried out to GMP standards. One significant limitation of the current method is the need for prolonged T cell cultures for the generation of the antigen-specific T cells. Preliminary experiments have been undertaken to optimize culture conditions, and to isolate (using FACSorting of HLA-peptide-pentamer stained cells) and expand antigen-specific CTLs *in vitro*, to try to reduce this time period.

## Conclusion

Our study demonstrates the ability of hybrid cell lines, formed by fusion of HMy2 cells and myeloma cells, to induce antigen specific cytotoxic T cells *in vitro* among PBLs obtained from HLA A2^+^ patients with multiple myeloma. The tumor antigen-specific T cells expanded, were cytotoxic and showed IFN-γ-releasing activity against TAAs expressed by myeloma cells. Interestingly, some of the TAAs used, such as hTERT and MUC1, are widely expressed in various tumors, suggesting that antigen-specific CTLs stimulated by the hybrid cell lines may not be exclusive to MM. It is noteworthy that HLA-A2 is of critical importance for restriction of antigen specific cytotoxic T lymphocytes recognition in tumor immunity (42). The loss of expression of this allele from tumor cells may be explained by the escape mechanism from recognition by T cells, which suggests its significant role in tumor immunosurveillance (43). Moreover, the frequency of HLA-A2 gene expression is high among different ethnic groups (44). Consequently, HLA-A2-restricted CTL epitopes might be valuable for the immunotherapy of many diseases all over the world.

## Ethics Statement

With respect to the involvement of human participants, the study was approved by the NRES Committee East Midlands—Northampton, study number 05/Q2502/26, and the University Hospitals of Leicester NHS Trust Research and Development Directorate, study number UHL 09708. All participants gave signed, informed consent. All procedures performed in studies involving human participants were conducted in accordance with the ethical standards of the institutional and/or national research ethics committee, and as laid down in the 1964 Declaration of Helsinki and its later amendments or comparable ethical standards.

## Author's Note

Parts of this paper has been published previously as part of the first author's doctoral thesis, *in vitro* generation of cytotoxic T cells with potential for adoptive tumor Immunotherapy, WK, Department of Infection, Immunity and Inflammation, University of Leicester, 2017 (45).

## Author Contributions

WK performed the experimental analysis. MG was responsible for recruitment of patients and anonymising data. YM contributed to the interpretation of data. CS co-supervised and supported the study and co-wrote the manuscript. MB conceived the project, directed the work, and assembly of the manuscript.

### Conflict of Interest Statement

The authors declare that the research was conducted in the absence of any commercial or financial relationships that could be construed as a potential conflict of interest.

## Supplementary Material

Supplementary Table 1 Patient characteristics from whom the starting material for CTL culture was obtained (time of study: 2015–2016; NBD, no paraprotein band detected in serum).

Supplementary Table 2 Numbers of PBMCs after each stimulation in a representative example of one stimulation experiment.

Supplementary Figure 1 Percentages of positive MUC1, h TERT, CS1, MAGE-C1 and WNF HLA-A^*^201 restricted, PE peptide pentamer-stained CTL clones. PBMCs isolated from four HLA A2- patients with MM were stimulated *in vitro* for 4 weeks using the hybrid cell lines HRC and HU266, or the parent cell lines HMY2 and U266 as stimulators. The cultures were stained with HLA-A2-peptide pentamers incorporating cognate peptide antigen form MUC 1, hTERT, CS1, MAGE-C1, or the irrelevant WNF peptide, followed by flowcytometric analysis. Responses of individual cell lines are shown on the X-axis. Percent of pentamer-positive cells is shown on the Y-axis. Results are presented as mean ± SEM.

Supplementary Figure 2 IFNγ ELISpot assays. Responder cells (PBMCs, isolated from three different HLA-A2- MM patients and stimulated with HMY2, HU266, HRC or U266) were mixed with T2 stimulator cells after pulsing with MUC1, h TERT, CS1, MAGE-C1 or WNF derived peptides) in IFNγ ELISpot assays. Responder:stimulator cell ratios are shown on the X-axis. Number of elicited spots is shown on the Y-axis. Data are presented as mean value of the patients' responses ± SEM. Stimulated PBMCS in the long-term culture of the used cell lines without further stimulation were used as a negative control in each corresponding ELISpot assay of the same cell lines. (There were insufficient cells in the cultures to perform perforin Elispot assays).

Supplementary Figure 3 IFNγ releasing ELISpot assay in one of HLA-A2 negative patient. There are observed increases of IFNγ releasing responses in the case of MUC1 (in all of the used ratios), h TERT (in 1:100 and 1:50), and CS1 only in 1:100 ratio **(A)**. Flowcytometric histograms of HLA-A2 expression by the HLA-A2 negative patient (in the upper row), and one of the HLA-A2 positive patients in the lower row. The left hand histogram of each line represents the isotype control staining of this patient, and the right hand histogram represents cells stained with the HLA-A2 specific mAb BB7.2 **(B)**. Flow cytometric analysis of the stability of HLA-A2 molecule of T2 cell line pulsed with the examined MM antigens, irrelevant WNF peptide antigen, or unpulsed T2 cell line **(C)**. Stability of HLA-A2 pulsed with WNF, MUC1, h TERT, CS1 and MAGE-C1 using flow cytometric analysis (MFI of HLA-A2 expression on T2 pulsed peptide) and Syfpeithi score **(D)**.

Supplementary Figure 4 The cytotoxic activity of the long-term stimulated antigen specific T lymphocytes. PBMCs isolated from three different HLA-A2- MM patients, were stimulated *in vitro* as above, and directed against T2 cell line pulsed with the relevant HLA-A2 restricted TAA-derived peptides of MUC1, h TERT, CS1 and MAGE-C1, or an irrelevant antigen peptide (WNF virus). K562 cells were used as NK activity controls. Responder:stimulator cell ratios are shown on the X-axis. Percent specific cytolysis is shown on the Y-axis. Data are presented as mean ± SEM of % specific lysis. No statistically significant responses were seen in any of the assays compared with negative controls.

## Abbreviations

APC: antigen presenting cells
ASCTL: antigen-specific, cytotoxic T-lymphocytes
B-LCL: B-lymphoblastoid cell line
CTL: cytotoxic T-lymphocytes
EBV: Epstein Barr virus
GMP: good manufacturing practice
MM: multiple myeloma
PBLs: peripheral blood lymphocytes
TAA: tumor associated antigen.
