# Supplementary material for: In vitro Generation of Cytotoxic T Cells With Potential for Adoptive Tumor Immunotherapy of Multiple Myeloma
Source: Front Immunol. 2019 Aug 2;10:1792. doi: 10.3389/fimmu.2019.01792 (PMC6687956; doi:10.3389/fimmu.2019.01792)
Supplement: Supplementary Table 1 — Patient characteristics from whom the starting material for CTL culture was obtained (time of study: 2015–2016; NBD, no paraprotein band detected in serum). [file Table_1.DOCX]

Suppl.Table 1

Patient characteristics from whom the starting material for CTL culture was obtained (time of study: 2015-2016; NBD, no paraprotein band detected in serum)

| **Pt no.** | **Sex** | **Age** | **Paraprotein**  **isotype** | **Paraprotein**  **Level**  (g/L) | **Current status** | **Current medication (myeloma)** | **Lymphocyte count**  (x10*9/L) | **HLA-A2 status** |
| --- | --- | --- | --- | --- | --- | --- | --- | --- |
| **1** | M | 67 | IgGκ | NBD | Complete remission | No current medication | 2.48 | negative |
| **2** | M | 67 | IgGκ | 27.7 | Stable myeloma | No current medication | 1.35 | positive |
| **3** | M | 75 | IgAκ | NBD | 2^nd^relapse 2015 | Lenalidomide/  Dexamethasone | 0.90 | negative |
| **4** | F | 71 | IgGλ | 8.6 | Stable myeloma | No current medication | 8.95 | positive |
| **5** | F | 86 | IgGκ | 26.1 | 3^rd^relapse 2016 | Stopped Lenalinomide March2016 (no response) | 1.08 | positive |
| **6** | M | 61 | IgAκ | NBD | 2^nd^relapse 2015 | Lenalinomide/ Dexamethasone | 2.29 | negative |
| **7** | F | 61 | IgAλ | NBD | Plasmacytoma “watch and wait” | No current medication | 1.29 | positive |
| **8** | M | 76 | IgGκ | 14.7 | 1^st^relapse 2016 | No current medication | 1.14 | negative |
| **9** | M | 54 | IgGκ | 6.4 | Stable remission | No current medication | 2.46 | positive |
