# Supplementary material for: In vitro Generation of Cytotoxic T Cells With Potential for Adoptive Tumor Immunotherapy of Multiple Myeloma
Source: Front Immunol. 2019 Aug 2;10:1792. doi: 10.3389/fimmu.2019.01792 (PMC6687956; doi:10.3389/fimmu.2019.01792)
Supplement: Supplementary Table 2 — Numbers of PBMCs after each stimulation in a representative example of one stimulation experiment. [file Table_2.DOCX]

|  | HMY2 X106 | HU266 X106 | HRC X106 | HIC X106 | U266 X106 |
| --- | --- | --- | --- | --- | --- |
| Zero | 2 | 2 | 2 | 2 | 2 |
| 1^st^ | 1.7 | 2.5 | 5.6 | 6.4 | 2.8 |
| 2^nd^ | 1.4 | 4.7 | 2.4 | 1.1 | 1.8 |
| 3^rd^ | 3.8 | 6 | 3 | 2.4 | 3 |
| 4^th^ | 4.8 | 8.5 | 4.5 | 2.25 | 2.8 |

Suppl Table 2: numbers of PBMCs after each stimulation in a representative example of one stimulation experiment.
