# Supplementary figures and images for: In vitro Generation of Cytotoxic T Cells With Potential for Adoptive Tumor Immunotherapy of Multiple Myeloma
Source: Front Immunol. 2019 Aug 2;10:1792. doi: 10.3389/fimmu.2019.01792 (PMC6687956; doi:10.3389/fimmu.2019.01792)

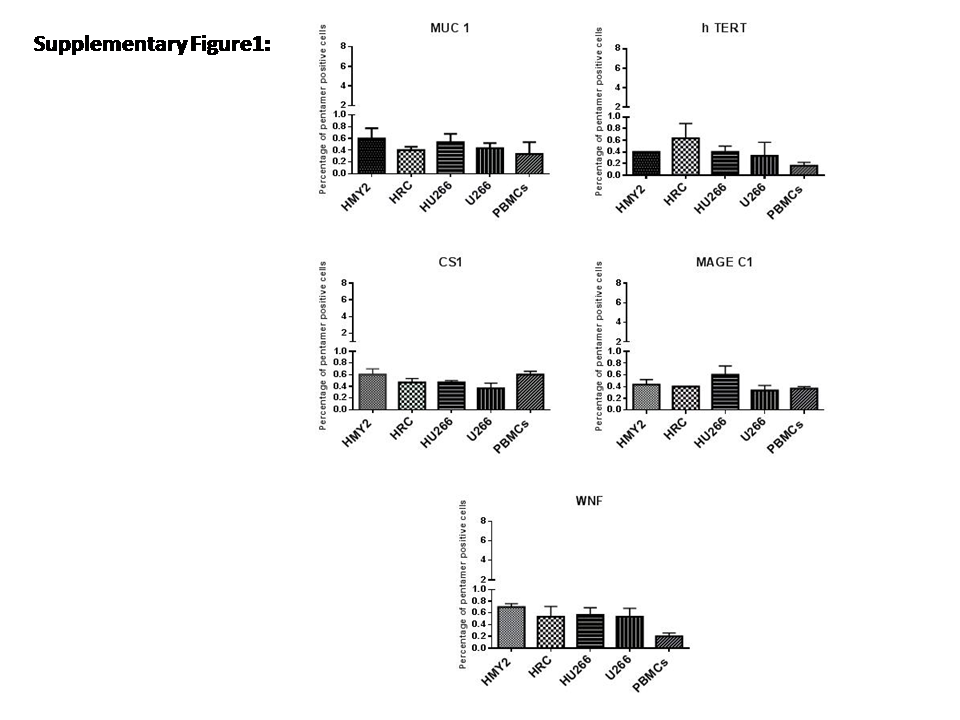

Supplement: Supplementary Figure 1 — Percentages of positive MUC1, h TERT, CS1, MAGE-C1 and WNF HLA-A*201 restricted, PE peptide pentamer-stained CTL clones. PBMCs isolated from four HLA A2- patients with MM were stimulated in vitro for 4 weeks using the hybrid cell lines HRC and HU266, or the parent cell lines HMY2 and U266 as stimulators. The cultures were stained with HLA-A2-peptide pentamers incorporating cognate peptide antigen form MUC 1, hTERT, CS1, MAGE-C1, or the irrelevant WNF peptide, followed by flowcytometric analysis. Responses of individual cell lines are shown on the X-axis. Percent of pentamer-positive cells is shown on the Y-axis. Results are presented as mean ± SEM. [file Image_1.TIF]

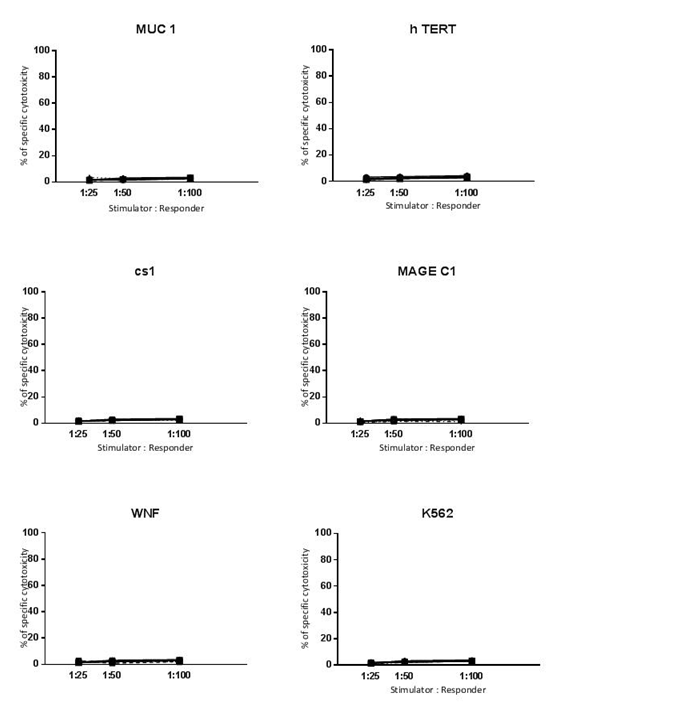

Supplement: Supplementary Figure 4 — The cytotoxic activity of the long-term stimulated antigen specific T lymphocytes. PBMCs isolated from three different HLA-A2- MM patients, were stimulated in vitro as above, and directed against T2 cell line pulsed with the relevant HLA-A2 restricted TAA-derived peptides of MUC1, h TERT, CS1 and MAGE-C1, or an irrelevant antigen peptide (WNF virus). K562 cells were used as NK activity controls. Responder:stimulator cell ratios are shown on the X-axis. Percent specific cytolysis is shown on the Y-axis. Data are presented as mean ± SEM of % specific lysis. No statistically significant responses were seen in any of the assays compared with negative controls. [file Image_4.tif]
