# Supplementary material for: In vitro Generation of Cytotoxic T Cells With Potential for Adoptive Tumor Immunotherapy of Multiple Myeloma
Source: Front Immunol. 2019 Aug 2;10:1792. doi: 10.3389/fimmu.2019.01792 (PMC6687956; doi:10.3389/fimmu.2019.01792)
Supplement: Supplementary Figure 3 — IFNγ releasing ELISpot assay in one of HLA-A2 negative patient. There are observed increases of IFNγ releasing responses in the case of MUC1 (in all of the used ratios), h TERT (in 1:100 and 1:50), and CS1 only in 1:100 ratio (A). Flowcytometric histograms of HLA-A2 expression by the HLA-A2 negative patient (in the upper row), and one of the HLA-A2 positive patients in the lower row. The left hand histogram of each line represents the isotype control staining of this patient, and the right hand histogram represents cells stained with the HLA-A2 specific mAb BB7.2 (B). Flow cytometric analysis of the stability of HLA-A2 molecule of T2 cell line pulsed with the examined MM antigens, irrelevant WNF peptide antigen, or unpulsed T2 cell line (C). Stability of HLA-A2 pulsed with WNF, MUC1, h TERT, CS1 and MAGE-C1 using flow cytometric analysis (MFI of HLA-A2 expression on T2 pulsed peptide) and Syfpeithi score (D). [file Image_3.pdf]

Supplementary figure 3

A

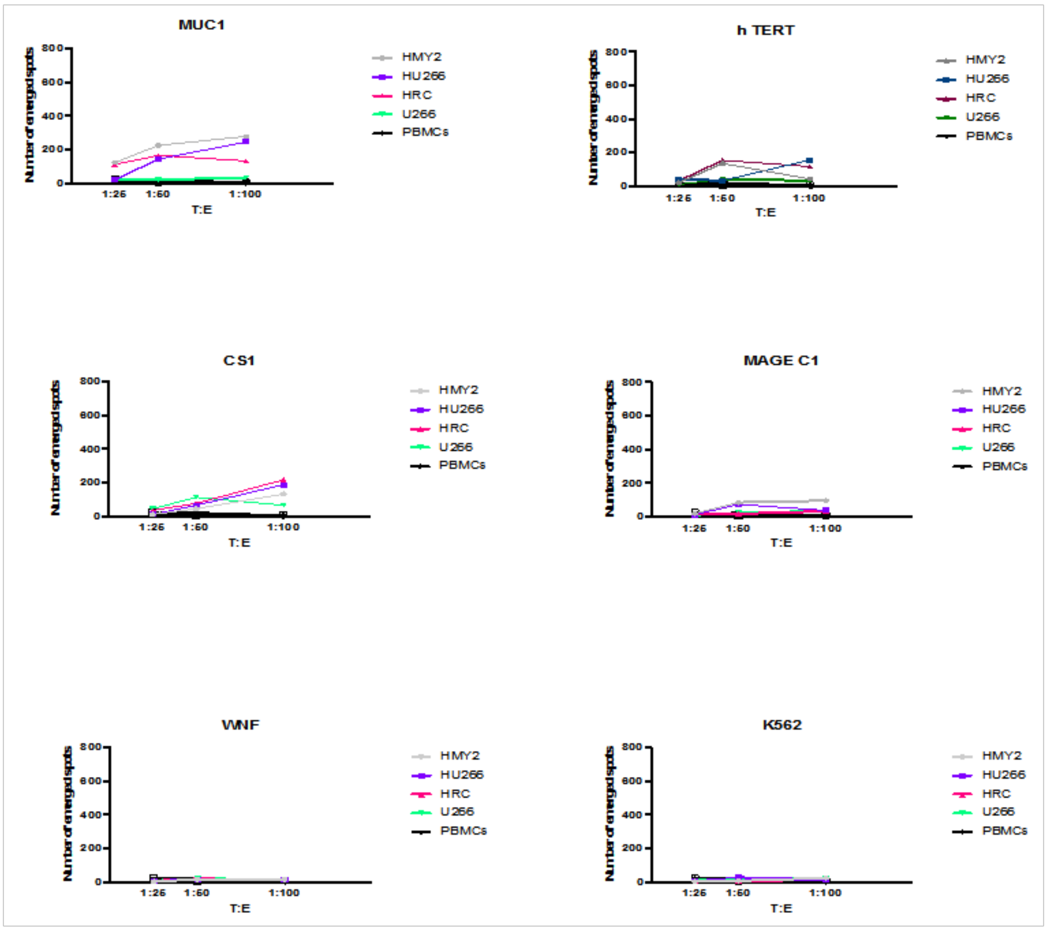

B

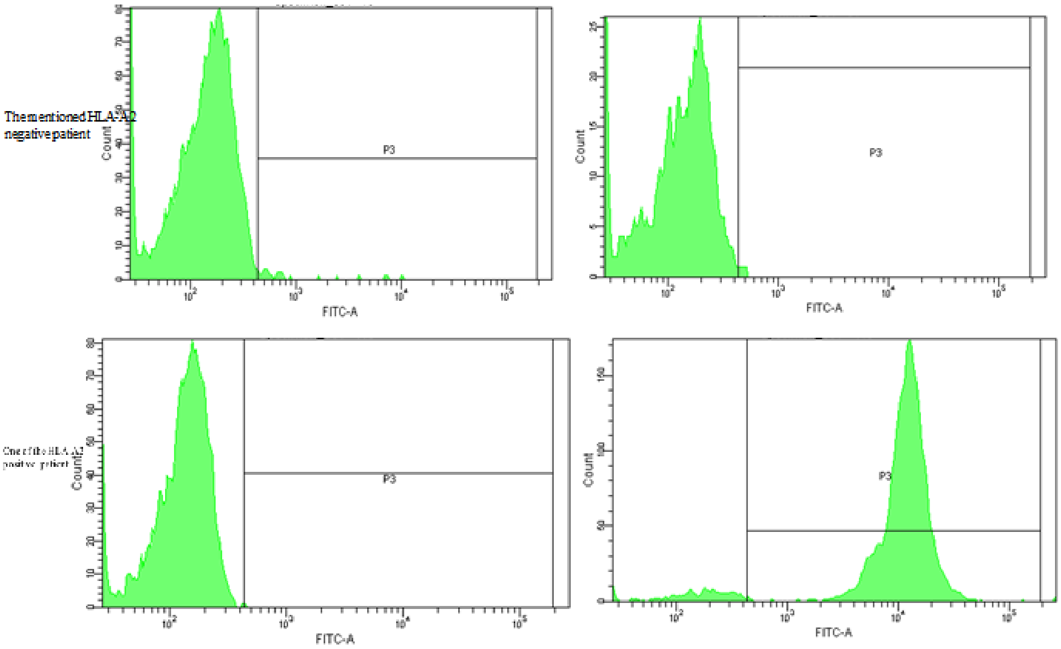

C

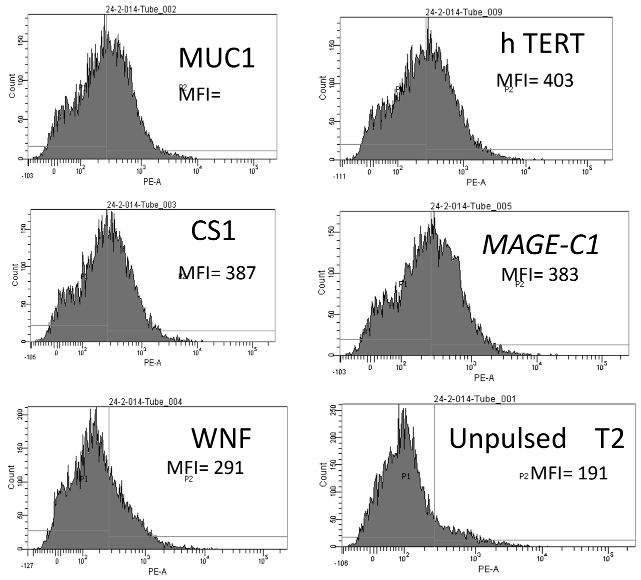

D

**Antigen**	**Peptide sequence**	**MFI after loading on T2 cells**	**Binding predictionscore(Syfpeithi score)**
**MUC1**	LLLLTVLTV	394	31
**hTERT**	ILAKFLHWL	403	30
**CS1**	SLFVLGLFL	387	25
MAGE-C1	ILFGISLREV	383	18
**WNF**	LGMSNRDFL	291	13
**UnpulsedT2cells**	N/A	191	N/A
